# MMP20-generated amelogenin cleavage products prevent formation of fan-shaped enamel malformations

**DOI:** 10.1038/s41598-021-90005-z

**Published:** 2021-05-19

**Authors:** John D. Bartlett, Charles E. Smith, Yuanyuan Hu, Atsushi Ikeda, Mike Strauss, Tian Liang, Ya-Hsiang Hsu, Amanda H. Trout, David W. McComb, Rebecca C. Freeman, James P. Simmer, Jan C.-C. Hu

**Affiliations:** 1 Division of Biosciences, Ohio State University College of Dentistry, Columbus, OH USA; 2 Department of Biologic and Materials Sciences, University of Michigan School of Dentistry, 1210 Eisenhower Pl, Ann Arbor, MI 48108 USA; 3 Department of Anatomy and Cell Biology, Faculty of Medicine and Health Sciences, McGill University, Montreal, QC Canada; 4 Department of Materials Science and Engineering, Ohio State University College of Engineering, Columbus, OH USA; 5 Center for Electron Microscopy and Analysis, Ohio State University, Columbus, OH USA

**Keywords:** Developmental biology, Structural biology

## Abstract

Dental enamel forms extracellularly as thin ribbons of amorphous calcium phosphate (ACP) that initiate on dentin mineral in close proximity to the ameloblast distal membrane. Secreted proteins are critical for this process. *Enam*^−/−^ and *Ambn*^−/−^ mice fail to form enamel. We characterize enamel ribbon formation in wild-type (WT), *Amelx*^−/−^ and *Mmp20*^−/−^ mouse mandibular incisors using focused ion beam scanning electron microscopy (FIB-SEM) in inverted backscatter mode. In *Amelx*^−/−^ mice, initial enamel mineral ribbons extending from dentin are similar in form to those of WT mice. As early enamel development progresses, the *Amelx*^−/−^ mineral ribbons develop multiple branches, resembling the staves of a Japanese fan. These striking fan-shaped structures cease growing after attaining ~ 20 µm of enamel thickness (WT is ~ 120 µm). The initial enamel mineral ribbons in *Mmp20*^−/−^ mice, like those of the *Amelx*^−/−^ and WT, extend from the dentin surface to the ameloblast membrane, but appear to be fewer in number and coated on their sides with organic material. Remarkably, *Mmp20*^−/−^ mineral ribbons also form fan-like structures that extend to ~ 20 µm from the dentin surface. However, these fans are subsequently capped with a hard, disorganized outer mineral layer. Amelogenin cleavage products are the only matrix components absent in both *Amelx*^−/−^ and *Mmp20*^−/−^ mice. We conclude that MMP20 and amelogenin are not critical for enamel mineral ribbon initiation, orientation, or initial shape. The pathological fan-like plates in these mice may form from the lack of amelogenin cleavage products, which appear necessary to form ordered hydroxyapatite.

## Introduction

Enamel formation is characterized by defined stages of development, which are arranged in the continuously growing rodent incisor from earliest (apical/posterior) to latest (incisal/anterior)^1^. In the anterior portion of the apical loop a basement membrane separates actively proliferating epithelial and mesenchymal layers^2^. Moving anterior from the apical loop, differentiating odontoblasts initiate secretion of mantle predentin composed primarily of type-I collagen^3^. Predentin is secreted adjacent to less-differentiated ameloblasts that become increasingly columnar and polarized^4^. As ameloblasts continue to differentiate, they begin secreting amelogenin^5^ and extend finger-like projections through the thinning basement membrane and into the predentin between collagen fibers^6^. As this process proceeds, an increasing proportion of ameloblasts extend cell processes through the thinning basement membrane. The remaining membrane fragments are endocytosed by ameloblasts and subsequently degraded by lysosomes^7^. Small foci of mineralization occur in predentin within a few micrometers from the ameloblasts^8,9^. These islands of mineral expand and merge into a continuous layer of dentin that encases the predentin^9^. Ameloblast finger-like projections initiate the formation of enamel mineral ribbons primarily on the sides and tips of mineralized collagen fibrils to form the DEJ^8^. These initial ribbons are part of the interrod component of enamel^10^. The mineral ribbons elongate as the ameloblast finger-like projections retreat back from the mineralized collagen to the distal surface of the ameloblast. Interrod enamel ribbons continue growing from the ameloblast membrane specifically at the cell junctions producing interrod prongs around the ameloblast cell boundaries. This creates space for the forming distal part of the Tomes’ process that makes rod enamel^11–14^. Tomes’ processes have discrete rod and interrod enamel ribbon growth areas characterized by marked infoldings of the distal (rod) and proximal (interrod) cell membrane^15^. As the secretory stage progresses, the rod and interrod ribbons elongate as ACP while the deeper part of the ribbons further away from the mineralized front transitions into HAP^16^. Enamel rods stop elongating when the enamel layer reaches its full thickness with the ends of the enamel covered by a final thin layer of interrod-type enamel^12^. At this point, the ameloblasts shorten and transition into the maturation stage of development. Then they accelerate reabsorption of protease-cleaved enamel matrix protein fragments so that the mineral ribbons can expand in width and thickness to fully mineralize and harden the enamel layer^1,17,18^.

During the secretory stage of enamel development, amelogenin (AMEL), ameloblastin (AMBN), enamelin (ENAM) and matrix metalloproteinase-20 (MMP20, enamelysin) are secreted collectively into the forming enamel layer^19^. Severe enamel malformations occur in both humans and mice when individual genes encoding these proteins are ablated^20^. The secretory stage enamel proteins that accumulate are predominantly amelogenin cleavage products with smaller amounts of ameloblastin and enamelin cleavage products^21,22^. Recombinant MMP20 cleaved recombinant amelogenin and ameloblastin in vitro to generate all of their known in vivo cleavage products, demonstrating that MMP20 is the only matrix proteinase that cleaves enamel matrix proteins during the secretory stage^23–25^.

No enamel layer forms in *Ambn*^−/−^^26^ and *Enam*^−/−^^8^ mice, demonstrating that these proteins are essential for enamel ribbon formation. *Amelx*^−/−^ and *Mmp20*^−/−^ mice each have severe enamel hypoplasia^8,27^, but their enamel phenotypes are distinctly different than those of *Ambn*^−/−^ or *Enam*^−/−^ mice. With the advent of focused ion beam (FIB) scanning electron microscopy (SEM) the fine structure of enamel morphology has become easier to discern. The advantages of FIB-SEM are that it visualizes the surface of a block face in TEM-like resolution without having to cut an ultrathin section from the block. Additionally, it can generate serial images by gallium ion beam milling for computerized tomography. It was definitively demonstrated by FIB-SEM that although the absence of ameloblastin or enamelin expression eliminates enamel ribbon formation, knockout of amelogenin does not^8^.

Here we show that as in *Amelx*^−/−^ mice, absence of *Mmp20* activity does not block formation of the initial enamel mineral ribbons but does result in the production of a defective enamel layer that bears remarkable similarities to the abnormal enamel layer seen in *Amelx*^−/−^ mice. Previously we demonstrated that enamel from *Mmp20*^−/−^ mice delaminates from the dentin surface^28^. Here we describe dysplastic enamel ribbon formation and elongation in *Mmp20*^−/−^ mice and compare and contrast these findings with dysplastic enamel formation in *Amelx*^−/−^ mice. Based upon these results, we propose a novel theory for the roles of MMP20 and amelogenin in enamel formation.

## Materials and methods

### Mice used in study

All animals used in this study were housed in Association for Assessment and Accreditation of Laboratory Animal Care International (AAALAC)-accredited facilities and were treated humanely based on protocols approved by the Ohio State University and the University of Michigan Institutional Animal Care and Use Committees and were carried out in compliance with ARRIVE guidelines. Experimental protocols were designed along University and National Institutes of Health (NIH) guidelines for the humane use of animals. *Amelx*^−/−^ and *Mmp20*^−/−^ mice^28,29^ were separately backcrossed into C57BL/6 (wild-type, WT) mice for at least eight generations to obtain a homogeneous genetic background so that analyses would not be affected by genetics other than the assessed respective deleted genes.

### Real-time quantitative PCR (qPCR) analyses

Total RNA extracted from 5-day old mouse first molars was isolated using Direct-zol RNA Mini Preps (Zymo Research). Total RNA was reverse-transcribed and cDNA was quantified by qPCR with mRNA specific primers. Relative expression levels of *Amelx, Ambn and Enam* were calculated by the 2^−ΔΔCt^ method using *Gapdh* cycle threshold (Ct) value as the housekeeping gene control^30^.

**Table:** 

Gene name	Forward primer	Reverse primer
*Amelx*	CTCATCCTGGAAGCCCTGGTTAT	GGCTGCCTTATCATGCTCTGGTA
*Ambn*	ATGAAGGGCCTGATCCTGTTC	GTCTCATTGTCTCAAGGCTCAAA
*Enam*	CTTTGGGGGTCGTCCTTATTACTC	CCTCTGGGGGTGGGTCATC
*Gapdh*	ACTGGCATGGCCTTCCG	CAGGCGGCACGTCAGATC

### In situ hybridization

Continuously erupting maxillary incisors were from adult mice and first molars were from postnatal day 5 pups. Both were from wild-type C57BL/6 mice. Teeth were formalin fixed and decalcified in 150 mM NaCl/10% acetic acid and were paraffin embedded, sectioned and deparaffinized in xylene. The antisense mouse *Mmp20* probe and assay kit was from Advanced Cell Diagnostics (Newark, CA). In situ hybridization was performed according to the manufacturer’s protocol.

### Mouse incisor sample preparation for microscopy

As described previously^8^, C57BL/6 seven week old mice that were either wild-type, *Mmp20*^−/−^ or *Amelx*^−/−^, were deeply anesthetized and then transcardial perfused with 2.5% glutaraldehyde in 0.08 M sodium cacodylate buffer with 0.05% calcium chloride. Mandibles were dissected, soft tissue removed, and the labial bone over the incisors was removed. Postfixation in the described perfusion buffer was for 4–6 h at 4 °C. The samples were washed with 0.1 M sodium cacodylate buffer overnight. Whole hemi-mandibles were then lipid stained with 1% reduced osmium tetroxide for 2 h, washed in several changes of distilled water, dehydrated in an acetone gradient, embedded with epoxy resin, and cured in a 60 °C oven for 48 h. Hemi-mandibles were cross sectioned at 1 mm increments at levels 1–8 and the slices were glued onto plastic stubs. The "levels" described herein represent the linear distance starting at the tip of the apical loop and following the initially curved (~ 0.2 mm) path of the basement membrane, then the ameloblast predentin/dentin interface, and finally the DEJ to the incisal tip.

### Definition of levels corresponding to stages of enamel development

Level 1 (starting approximately 1 mm curve distance from the tip of the apical loop) corresponds to the beginning of the secretory stage, Level 3 is around the transition between the secretory and maturation stages, and Level 8 is fully mature enamel about to erupt into the oral cavity at the gingival margin along the labial side of the incisor^31^. Blocks from right incisors were faced and aligned across the transverse plane using glass knives on a Leica Ultracut E ultramicrotome. Blocks from left mandibular incisors were sawed from the mounting stubs and reoriented for cutting in the sagittal (longitudinal) plane. These blocks were also faced and oriented using glass knives.

### Focused ion beam scanning electron microscopy (FIB-SEM)

This method was described previously in detail^8^. Briefly, the glass-faced blocks were attached to 45° chamfered mounting stubs with conductive silver paste and placed into a Helios Nanolab 660 FIB-SEM (FEI, Systems for Research, Longueuil, QC, Canada). A sampling area 100 µm × 100 µm in size was selected and milled with gallium ions at rough (45 nA) followed by fine (9.4 nA) settings. Imaging was done with the through lens detector (TLD) and where possible with the in column detector (ICD). All FIB-SEM imaging was done using blocks prepared for sagittal (longitudinal) views of the enamel layer and associated enamel organ cells. Where possible, blocks from 3 different mice per genotype were examined. Charging on the surfaces of block faces was reduced by coating with a thin layer of platinum (3 nm) where required. Curtaining defects in TLD images were reduced using python language software modified from Schwartz et al.^32^.

### Small area electron diffraction (SAED) imaging and analyses

SAED imaging of the enamel layer from wild type, *Mmp20*^−/−^ and *Amelx*^−/−^ mice was done in the postsecretory transition to early maturation region (between Levels 3–4) using blocks prepared in the transverse plane of the incisor for preparing thin sections cut at 85 nm on a ultramicrotome and mounted on 300 mesh grids, and blocks prepared in the sagittal plane for FIB-SEM milling at 90° into the block faces thereby producing thin lamella of about 100 nm thickness that were also then viewed in a transverse plane by TEM^33^. Electron diffraction imaging was done in a Tecnai F20 transmission electron microscope (FEI, Systems for Research, Longueuil, QC, Canada) equipped with TVIPS XF416 camera (Tietz Video and Image Processing Systems, Eremitenweg 1, D-82131, Gauting, Germany) using SerialEM protocol for acquisition^34^. Diffraction patterns were collected by locating and centering a feature of interest at × 7,800, inserting a 40 µm selected area aperture and collecting SAED patterns at a nominal camera length of 520 mm. Imaging in wild-type mice was done over dentin located near the DEJ and over single spots within the inner, middle, and outer thirds of the enamel layer. Imaging in *Mmp20*^−/−^ enamel was done near the DEJ, within the core of the mineral fans and in the outer disorganized layer at the surface. Imaging in *Amelx*^−/−^ enamel was done near the DEJ, within the stalk of the fans and within the body of the fans. These sites of sampling are illustrated in Fig. 8. Mineral phase differentiation was done by comparing the experimental SAED patterns to calculated electron diffraction patterns of various reference standards. Reference standards from the Inorganic Crystallographic Structure Database (https://icsd.nist.gov/) that were used included: hydroxyapatite (ICSD-26204), calcium polyphosphate (ICSD-60117), monoclinic hydroxyapatite (ICSD-34457) and octacalcium phosphate (ICSD-65347). Simulated electron diffraction patterns of these standards were calculated using version 10.5.7 of CrystalMaker and version 4.1.1 of Single Crystal Software (CrystalMaker Software Limited, Woodstock Road Begbroke Oxfordshire, OX5 1PF, UK; http://www.crystalmaker.com/software/index.html). Details about some of these analyses are presented in the Supplemental Data (S3–S6).

## Results

### ***Amelx, Ambn,*** and ***Enam*** transcript levels are normal in ***Mmp20***^−/−^ mice

**Figure 1:**
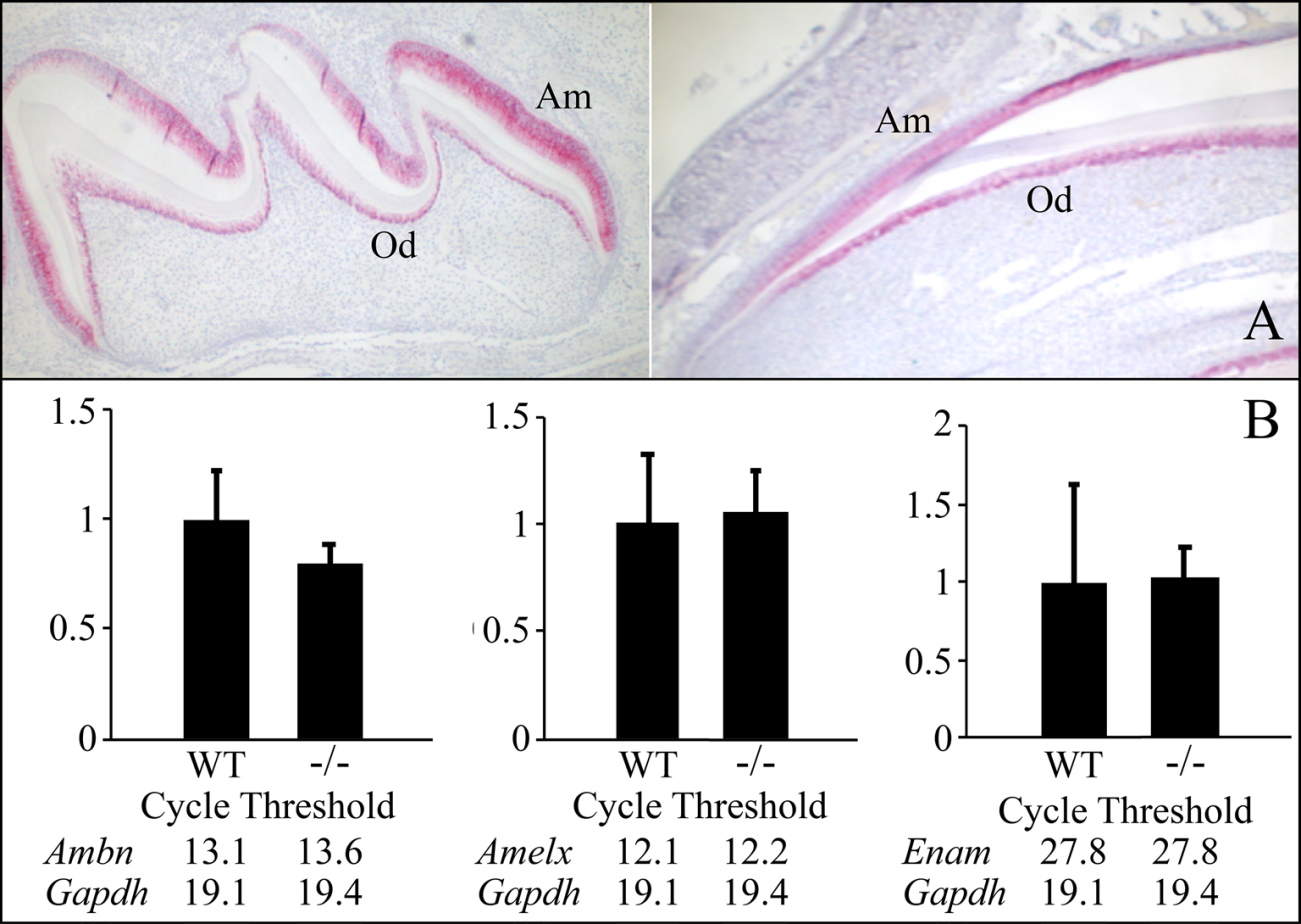
Wild-type (WT) and *Mmp20*^−/−^ mice express similar amounts of enamel matrix protein transcripts. The red staining from *Mmp20 *in situ hybridization (**A**) in a wild-type mouse Day-5 first molar (left) and maxillary incisor (right) confirms that both ameloblasts (Am) and odontoblasts (Od) normally express *Mmp20.* (**B**) qRT-PCR of mRNA isolated from postnatal Day-5 first molar enamel organs shows no difference in *Ambn*, *Amelx,* and *Enam* mRNA levels relative to the *Gapdh* housekeeping control in WT versus *Mmp20*^−/−^ mice.

We confirmed by in situ hybridization that wild-type *Mmp20* is expressed in ameloblasts and odontoblasts (Fig. 1A). We also demonstrate by quantitative real-time PCR (qPCR) that mRNA transcript levels for *Ambn*, *Amelx*, and *Enam* are not significantly different between WT and *Mmp20*^−/−^ mouse molars (Fig. 1B). Since this study addresses the relative contribution of MMP20 and its enamel matrix cleavage products to enamel formation, we confirm that in the absence of MMP20 other enamel matrix proteins are still transcribed and secreted into the enamel matrix. Both protein gels^28^ and immunoblots^35^ have demonstrated that enamel matrix proteins are secreted at similar levels in wild-type (WT) and *Mmp20*^−/−^ mice. Therefore, the enamel phenotype in *Mmp20*^−/−^ mice is not due to secondary effects from altered expression enamel matrix proteins.

### Ameloblasts extend finger-like projections to penetrate the basement membrane and this occurs in ***Amelx***^−/−^ and ***Mmp20***^−/−^ mice

**Figure 2:**
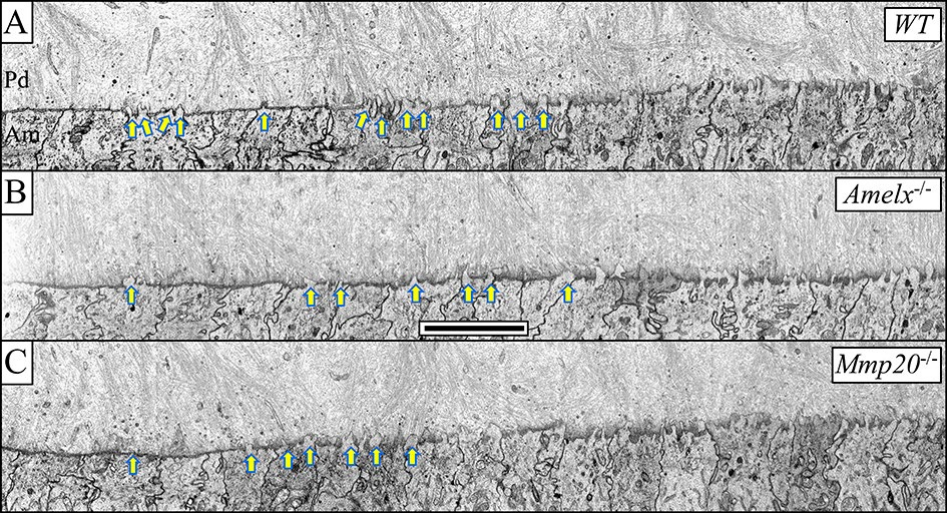
Pre-ameloblasts (Level 0.73) from WT, *Amelx*^−/−^, and *Mmp20*^−/−^ mice all penetrate the basement membrane that separates them from predentin. (**A**) Disruption of the wild-type basement membrane, separating differentiating ameloblasts (Am) from predentin, (Pd) is initiated by small finger-like projections from the distal surfaces of ameloblasts penetrating through the lamina densa of the membrane (yellow arrows). This process is initiated over several more widely spaced ameloblasts within the sagittal plane. Invaginations form between the finger-like projections engulf basement membrane components that are transported to lysosomes for disposal. For (**B**) *Amelx*^−/−^ and (**C**) *Mmp20*^−/−^ mice, a similar sequence of events occurs where thin finger-like processes from differentiating ameloblasts penetrating the basement membrane but, in this *Mmp20*^−/−^ example (**C**), over a somewhat shorter sagittal length compared to wild-type (3 in this case). Bar 5 µm for all panels.

In wild-type mice, disruption of the basement membrane between pre-ameloblasts and pre-odontoblasts occurs near the apical end of the incisor (Level 0.73, see “Materials and methods” for level definitions). Ameloblasts extend finger-like processes from their distal surface that breach the thinning basement membrane. As development proceeds, increasing numbers of these ameloblast processes penetrate the basement membrane and extend a short distance into predentin (Fig. 2A, yellow arrows). A similar sequence of events occurs in *Amelx*^−/−^ ameloblasts (Fig. 2B).

The basement membrane contains Type IV collagen^7,36^ and this collagen is an MMP20 substrate in vitro^37^. Therefore, we asked if MMP20 plays a role in ameloblast basement membrane penetration. We discovered that in the absence of MMP20, the basement membrane thins and is penetrated by ameloblast finger-like projections (Fig. 2C) as occurs normally in WT mice.

### Dentin mineralization in ***Amelx***^−/−^ and ***Mmp20***^−/−^ mice is similar to wild-type mice

**Figure 3:**
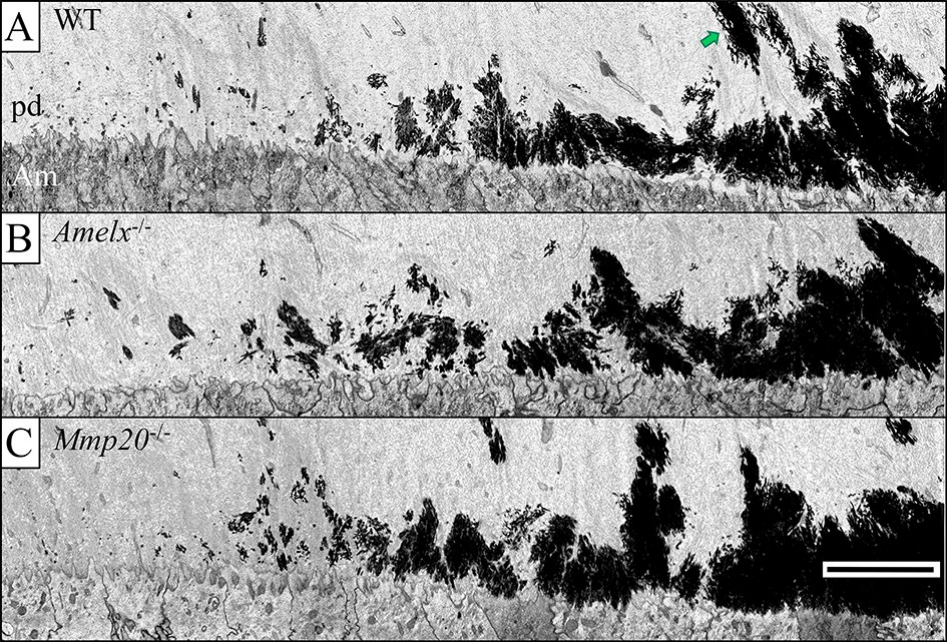
The pattern of dentin mineralization is similar in WT, *Amelx*^−/−^ and *Mmp20*^−/−^ mice. Backscatter FIB-SEM montages (5000 ×) of comparable longitudinally cut mandibular incisor blocks. The blocks cover the region of the incisor where mineralization first appears in mantle predentin (Level 0.9–1.0). (**A**) WT, initial mineralization of predentin (pd) does not occur as a solid front but initiates as isolated circular and elongated foci situated near differentiating ameloblasts. This occurs to a depth of about 3–5 µm into the predentin layer over a sagittal length of about 14 µm. Mineralization continues to spread in a sagittal direction over an additional 7 µm thereby solidifying a continuous mineralization front. From this point incisally, the mineralization front appears undulating with periodic mineralized extensions projecting toward the odontoblasts that extend along the length of large collagen fiber bundles (green arrow). For (**B**) *Amelx*^−/−^ and (**C**) *Mmp20*^−/−^ mice, the initial appearance of mineral in the predentin of *Amelx*^−/−^ and *Mmp20*^−/−^ incisors is similar to the WT. Bar 5 µm for all panels. *PD* predentin, *Am* ameloblast.

In WT (Fig. 3A), *Amelx*^−/−^ (Fig. 3B), and *Mmp20*^−/−^ (Fig. 3C) mice, dentin mineralization starts with small foci of mineral that generally initiate within several micrometers of the ameloblast layer near the eventual DEJ^8^. As these foci form, they grow in size and begin to merge with adjacent areas of mineralization. As this process proceeds, the area nearest the ameloblasts is the first to have a continuously mineralized dentin matrix. Periodic mineralized extensions also form that project toward the odontoblasts, and these extensions are typically associated with large collagen fiber bundles (Fig. 3A, green arrow). This same dentin mineralization process also occurs in *Amelx*^−/−^ and *Mmp20*^−/−^ mice (Fig. 3B,C). Although odontoblasts express MMP20 (Fig. 1), the steps leading to dentin mineralization in *Mmp20*^−/−^ mice also occur in an apparently normal fashion (Fig. 3C).

### A comparison of the initial enamel in WT, ***Amelx***^−/−^, and ***Mmp20***^−/−^ mice

**Figure 4:**
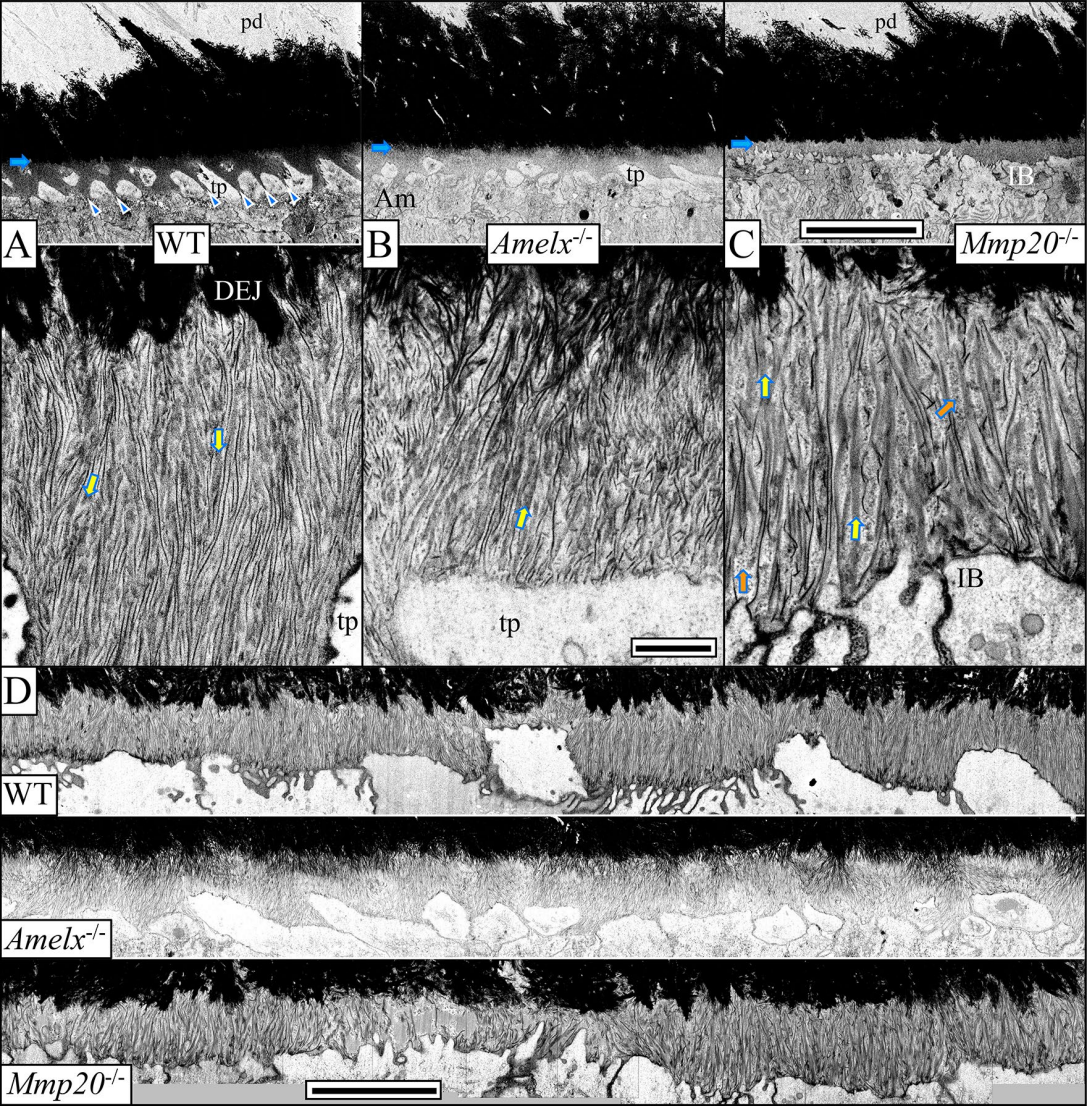
FIB-SEM micrographs of recently formed initial enamel (Level 1.2) in WT (**A**), *Amelx*^−/−^ (**B**) and *Mmp20*^−/−^ (**C**) mice. The top images (2500 ×, bar 10 µm) and bottom images (35,000 ×, bar 0.5 µm) are from the same specimen. From top to bottom the WT low magnifications show unmineralized predentin (pd), highly mineralized dentin (black), the DEJ (blue arrows), ameloblast Tomes' processes (tp). WT ameloblasts (Am) elongate prongs of interrod enamel along the membrane covering the intercellular junctions (blue arrowheads). The dark lines are believed to be the edges of enamel mineral ribbons and gray areas the broader, but thinner, sides (yellow arrows). *Amelx*^−/−^ ameloblasts make an abortive effort to create Tomes’ processes at their distal ends but fail to extend interrod prongs that ordinarily encase them and do not establish rod/interrod organization. They form thin, oriented enamel mineral ribbons, but the base of the ribbons take on a dark, bulkier appearance. *Mmp20*^−/−^ ameloblasts never establish the protruding portion of the Tomes process. The distal ends of ameloblasts form an irregular border (IB). *Mmp20*^−/−^ mineral ribbons appear to be fewer (yellow arrows). The inter-crystalline spaces appear larger compared to WT and are filled with small dot-like masses of material, likely representing uncleaved enamel proteins (orange arrows). Montages of high magnification images (**D**, bar 5 µm) in the sagittal plane show progression of enamel ribbon formation.

WT enamel mineral ribbons initiate on preexisting dentin mineral, predominantly on the sides and tips of mineralized collagen fibers at the DEJ, and are associated with ameloblast finger-like extensions^8^. These ribbons extend from their origins on mineralized collagen along the path that the finger-like processes take when they retract back to the ameloblast cell membrane (Fig. 4A)^8^. Oriented enamel mineral ribbons also form on the dentin surface of *Amelx*^−/−^ (Fig. 4B), *and Mmp20*^−/−^ (Fig. 4C) mice, but do not form on mice lacking enamelin^8^ or ameloblastin^26^.

Subtle differences in the initial mineral ribbons of WT, *Amelx*^−/−^, *and Mmp20*^−/−^ mice become more obvious as mineralization progresses. In contrast to WT ribbon growth (Fig. 4A), the *Amelx*^−/−^ enamel ribbons increase in density near the dentin surface when the ribbons are less than 3 µm in length (Fig. 4B,D). This increase in mineralization temporarily blurs the dentin-enamel boundary (Fig. 4B,D), and this results from the premature thickening of the mineral ribbons.

The *Mmp20*^−/−^ mineral ribbons appear as closely packed thin dark lines when seen on-edge or as broad, gray rectangular areas when seen in side view (Fig. 4C), presumably because they are coated with uncleaved amelogenins that adsorb onto mineral through their charged C-termini^38^, which MMP20 ordinarily cleaves soon after amelogenin secretion^39,40^. The binding of uncleaved amelogenins to the sides of the *Mmp20*^-/-^ initial mineral ribbons may explain why this layer remains *hypo*mineralized and later fails at the DEJ following eruption in *Mmp20*^−/−^ mice^20^. This is the exact opposite of the premature *hyper*mineralization of the initial enamel ribbons in the *Amelx*^−/−^ mice, that lack amelogenin.

### A comparison of appositional growth in WT, ***Amelx***^−/−^ and ***Mmp20***^−/−^ mice

**Figure 5:**
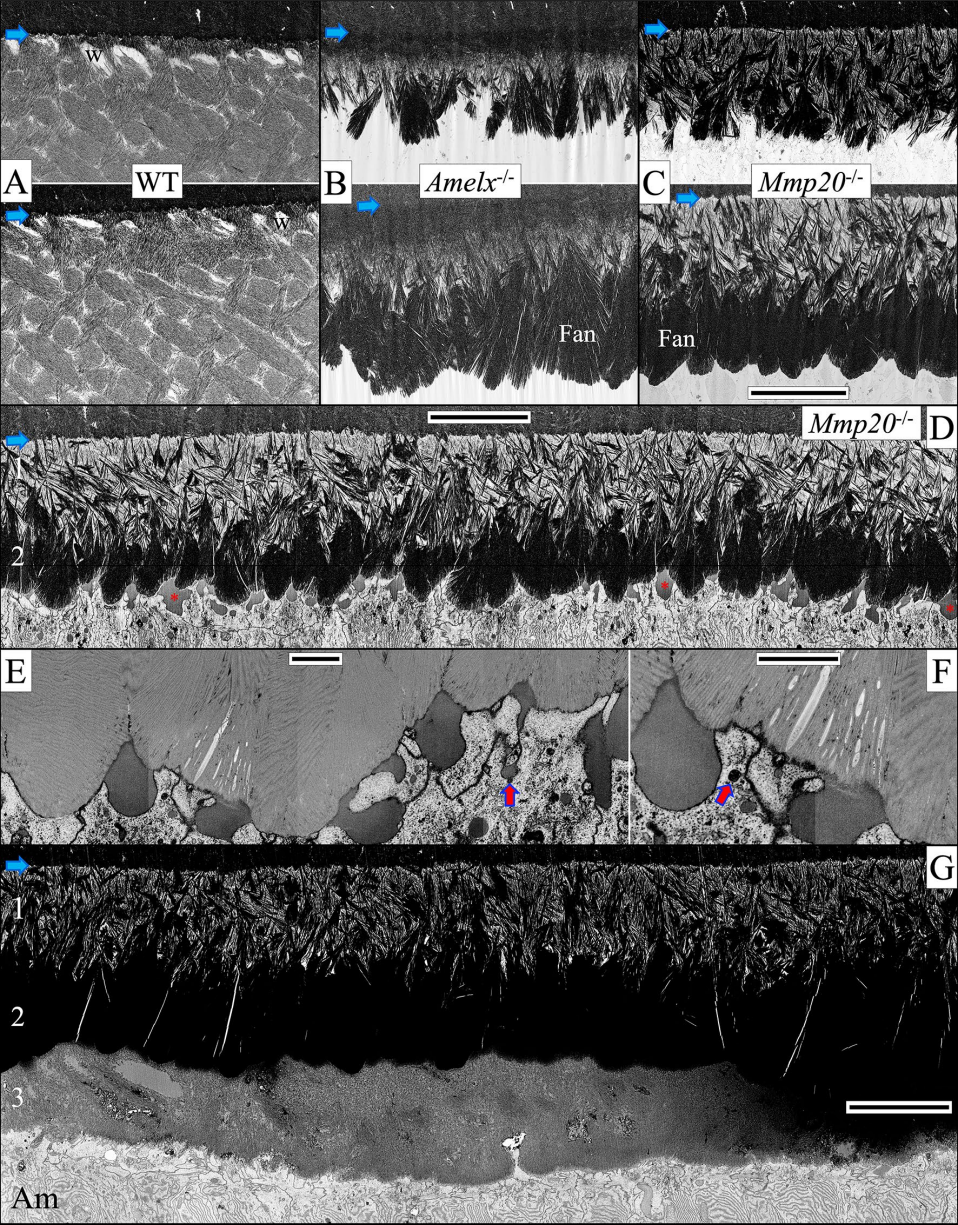
In contrast to WT enamel, *Amelx*^−/−^, and *Mmp20*^−/−^ enamel shows dysplastic mineralization in the form of fan-like structures (top row, Level 2, mid-secretion; bottom row, Level 3, end of secretory stage). Blue arrows mark the DEJ. The enamel layer in WT mice (**A**) increases through appositional growth to ~ 60 µm in thickness by the middle of the secretory stage (only the inner ~ 25 µm are shown) and terminates at ~ 120 µm at Level 3. This enamel typically presents as alternating rows of enamel rods as ovals in WT sagittal sections. The spaces of Weber (w) near the DEJ are clearly delineated. The dysplastic enamel layer in *Amelx*^−/−^ mice (**B**) at an equivalent distance from the tip of the apical loop is about 12 µm in thickness consisting of a dense inner layer and a lighter outer zone from which numerous dense fan-shaped structures grow and expand. The *Mmp20*^−/−^ dysplastic enamel layer (**C**) at a comparable location consists of a less dense inner layer comprised of smaller aborted fans. It also consists of the stems and initial branches of the fans that elongate to generate the second layer of larger fans that harden by filling-in the spaces between fan staves. At this point the dysplastic enamel bilayers in *Amelx*^−/−^ and *Mmp20*^−/−^ mice are the same thickness (~ 20–24 µm). The *Mmp20*^−/−^ late secretory stage, however, adds a third outer mineralized layer (**D**–**G**). This pathological layer does not form by the extension of mineral ribbons, but by ameloblasts rapidly secreting large quantities of protein a portion of which forms pools of protein extracellularly (red asterisks), on top of the already hardened fan structures. This outer layer is disorganized but sometimes shows patterns left by the retrograde movement of the ameloblast. Bars for panels (**A**–**D**), (**G**) = 10 µm, (**E**,**F**) = 1 µm.

**Figure 6:**
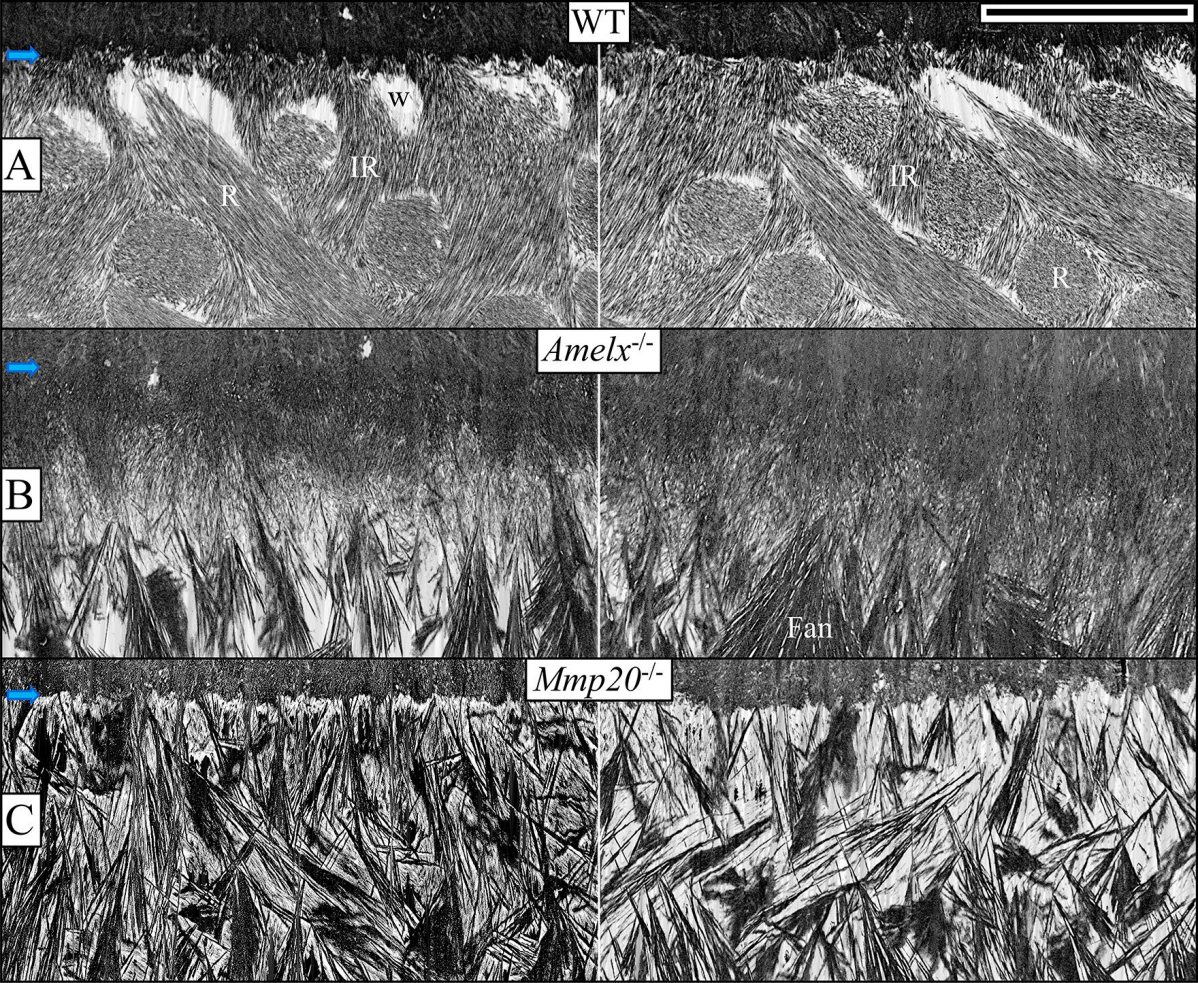
These images show a magnified view of WT, *Amelx*^−/−^, and *Mmp20*^−/−^ enamel in the region 8 µm away from the DEJ (blue arrows) midway through the secretory stage (Level 2, left column) and near the end of secretory stage (Level 3, right column). WT enamel (**A**) mineral ribbons are oriented and organized into rod (R) and interrod (IR) structures that have transitioned into HAP and are growing in width and thickness. At the base of the rods are spaces of Weber (w). *Amelx*^−/−^ enamel (**B**) becomes highly mineralized near the DEJ, but less mineralized in the stem region where most of the branching into fans occurs. *Mmp20*^−/−^ enamel (**C**) closest to the DEJ is severely hypomineralized and contains small micro fans. The larger fans that dominate the rest of the secretory stage start branching from mineral stems. This hypomineralized dysplastic enamel layer covering the DEJ explains why *Mmp20*^−/−^ enamel delaminates so readily at the DEJ (no shear/fracture resistance). Like *Amelx*^−/−^ enamel, *Mmp20*^−/−^ enamel has no enamel rod (R) or interrod (IR) organization. Bar 5 µm for all panels.

In WT mice, immediately following formation of the initial enamel, the end of each ameloblast reconfigures into a Tomes' process which, through two spatially distinct locations, organizes enamel mineral ribbons into rod and interrod structures^14^ (Fig. 4A). Rows of ameloblasts with Tomes' processes move in alternating directions, possibly through the use of contractile elements in ameloblasts^41^, to form the characteristic rodent decussating enamel rod patterns (Fig. 5A)^42^. By the end of the secretory stage, the mineral ribbons have progressively transitioned into HAP, progressively expanded in width and thickness with depth^43^, and reduced the prominence of the spaces of Weber at the base of the rods near the DEJ (Figs. 5A, 6A).

**Figure 7:**
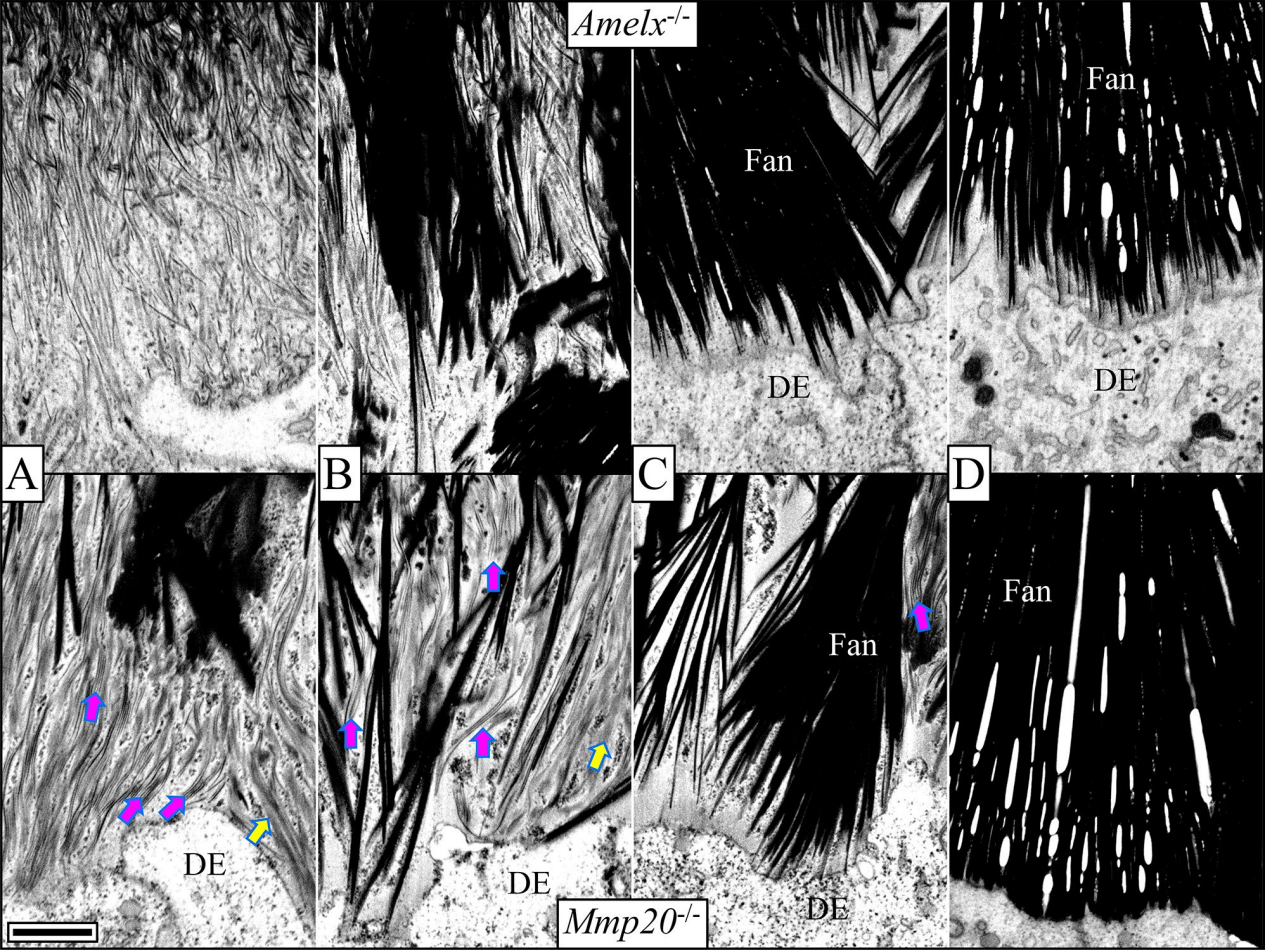
This series of high magnification (35,000 ×) micrographs show four secretory stage sites along the ameloblast distal membrane progressing from Level ~ 1.5 (**A**) to Level ~ 2.8 (**D**) in *Amelx*^−/−^ (top) and *Mmp20*^−/−^ (bottom) mouse incisors. At Level 1.5, thin enamel mineral ribbons extend along the mineralization front and are generally oriented in the direction from dentin surface to the ameloblast membrane. As before, the *Mmp20*^−/−^ ribbons appear with gray sides (yellow arrows), perhaps because they are coated with uncleaved enamel proteins, although other mineral ribbons are evident among the greyish lines (magenta arrows). The mineral ribbons transition into dense mineral crystals deep to the mineralization front. This apparent ribbon to crystal transition advances to the ameloblast membrane (**B**) and is associated with the termination of ribbon elongation and the thickening of the fans into plates (**C**,**D**). The *Amelx*^−/−^ and *Mmp20*^−/−^ mineral fans near the enamel surface are remarkably similar, suggesting they share important commonalities in their pathological mechanisms. *DE* distal end of ameloblasts. Bar 500 nm for all panels.

After forming the initial enamel, *Amelx*^−/−^ ameloblasts appear to form distal processes (Fig. 4D) and thereafter enamel mineralization deviates significantly from WT because the succeeding dysplastic enamel mineral layer lacks rod/interrod architecture and spaces of Weber. From the initial *Amelx*^−/−^ enamel layer emerges an unevenly mineralized second layer comprised at its base of mineralized stems and branching structures that resemble the staves of Japanese fans (Fig. 5B). These fan-like structures elongate and then thicken into mineral plates (Figs. 6B, 7A–D). The fans appear to arise through the formation of multiple mineral branches from a relatively thin, mineral stem (Fig. 6B).

**Figure 8:**
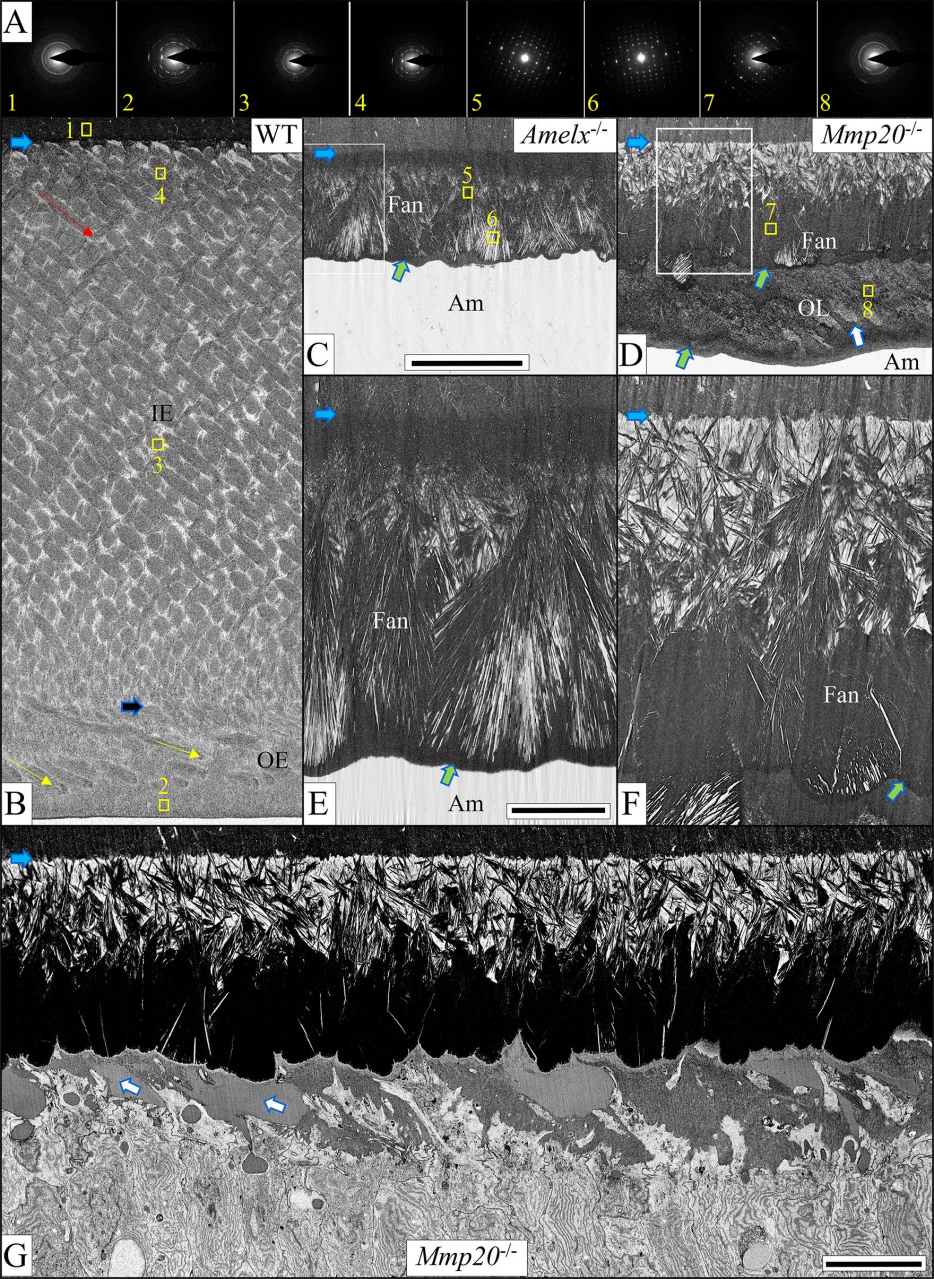
Early maturation stage (Level 4 +) enamel. Selected area electron diffraction (SAED) images (**A**) sampled at sites comparable to the small numbered yellow boxes shown in panels (**B**–**D**). Montages of focused ion beam-scanning electron micrographs (FIB-SEM) show early maturation stage enamel formed in WT (**B**), *Amelx*^−/−^ (**C**,**E**), and *Mmp20*^−/−^ (**D**,**F**) mice. WT enamel (**B**) is 120 µm thick on average and comprised of inner enamel (IE, 100 µm) with rods angled at approximately 42° (red arrow) to the horizontal plane of the DEJ (light blue arrows), and a thin outer enamel (OE, 20 µm) with rods angled at approximately 20° to the horizontal (yellow arrow). A dark blue arrow delineates the IE-OE border. *Amelx*^−/−^ enamel (**C**,**E**) is only ~ 20 to 24 µm thick, with two mineral layers: a thin (~ 5 µm) but highly mineralized inner layer covering the DEJ, covered by a thicker layer of mineral fans that is less mineralized in the stem region and more highly mineralized where the fans are extended. The tops of the fans are covered with a thin coating of evenly dense mineral (green arrows). *Mmp20*^−/−^ enamel (**D**,**F**,**G**) is ~ 40 µm thick and consists of 3 layers: a poorly mineralized inner layer, a middle layer formed by dense mineral fans also covered with a thin coating of evenly dense mineral (green arrows), and a disorganized, unevenly mineralized outer layer (OL). Occasionally poorly mineralized rod-like structures are observed in the superficial layer (white arrows), but a secretory stage montage showing this OL forming (**G**), shows no Tomes' processes or enamel mineral ribbons but only a disorganized mineral layer forming in the wake of retreating ameloblasts. This montage is intermediate between those shown in Fig. 5D,E. Bar in (**C**,**D**) = 20 µm, in (**E**,**F**) = 5 µm, in (**G**) = 10 µm.

**Figure 9:**
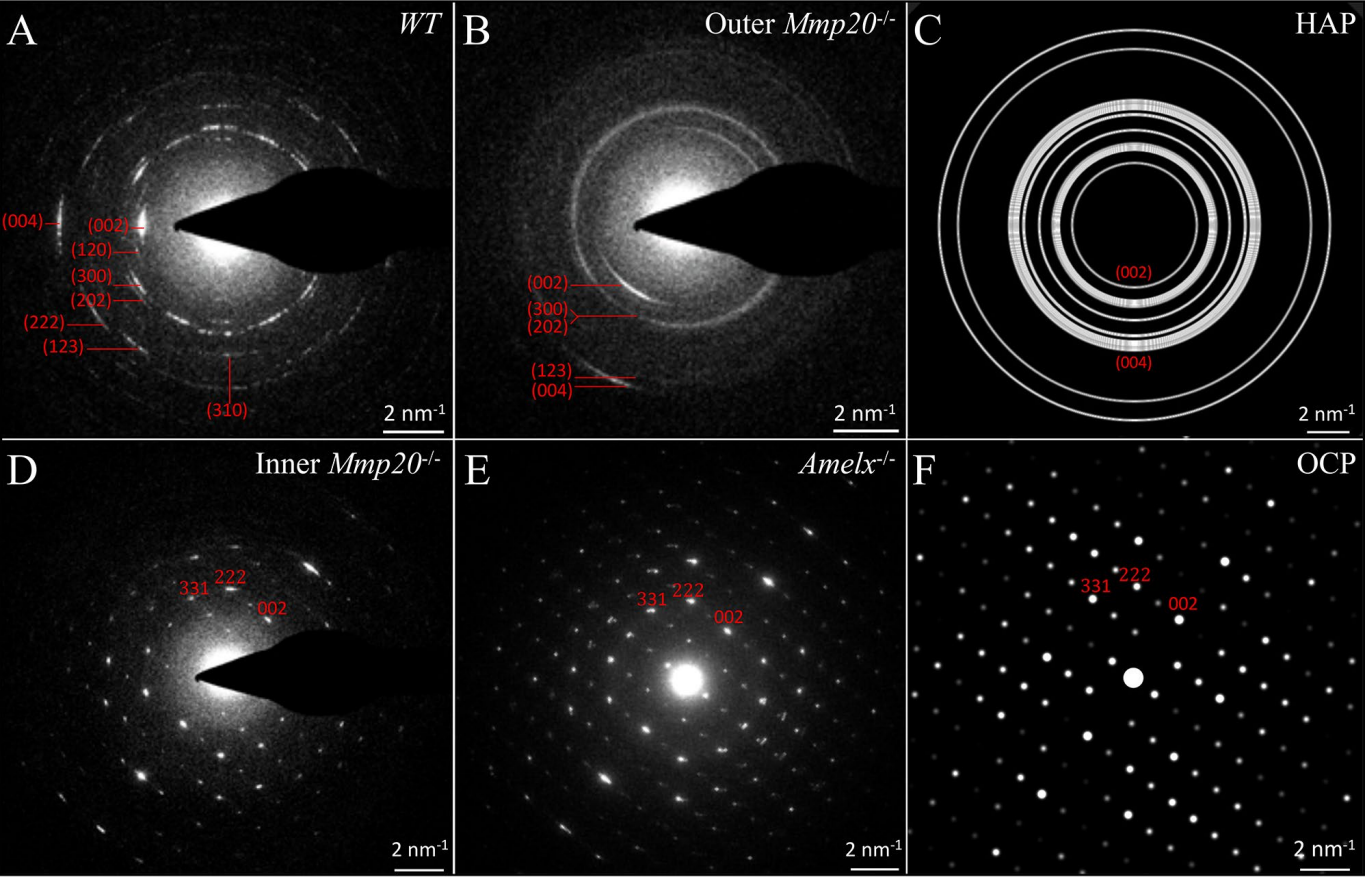
Mineral Phase Determination. The TEM diffraction patterns acquired from the outer enamel layer of WT mice (**A**) and outer enamel layer of *Mmp20* null mice (**B**) matched the calculated pattern of hydroxyapatite (**C**) (ICSD-26204), while the diffraction patterns acquired from a fan structure in the *Mmp20*^−/−^ inner enamel (**D**) and *Amelx*^−/−^ enamel (**E**) matched the calculated pattern of octacalcium phosphate (**F**) (ICSD-65347) following rotation into a common orientation.

Previously the crystalline phase associated with the fans in *Amelx*^−/−^ mice was shown to be octacalcium phosphate (OCP)^27^ and here we confirm this result (Fig. 8 diffraction patterns at top, Fig. 9, and Supplemental data Fig. S6). The fans are disorganized, and tilt at angles toward the ameloblast layer and overlap. As development proceeds, the tips of the OCP fan staves are in close proximity with the distal membrane of the ameloblasts and thicken and merge only a short distance away (Fig. 7, top).

Dysplastic enamel formation in *Mmp20*^−/−^ mice also leads to the development of poor-quality enamel that contains large fan-like structures (Fig. 5C). However, in contrast to *Amelx*^−/−^ mice, the mineral in *Mmp20*^−/−^ mice begins organizing almost immediately into “micro fans”, some of which branch at or near the DEJ (Fig. 6C). As in *Amelx*^−/−^ mice, the enamel mineral ribbons eventually thicken and apparently crystalize to form fan-like branches that grow to contact the distal membranes of ameloblasts (Fig. 7). At Level 3 the *Amelx*^−/−^ and *Mmp20*^−/−^ bilayers (~ 20–24 µm in thickness) are both comprised of a forest-like array of fan-shaped plates (Fig. 4A–C). SAED analyses indicate that the diffraction patterns for the fans in *Mmp20*^−/−^ mice are similar to diffraction patterns in *Amelx*^−/−^ mice and the crystal lattice spacing for both patterns matches that of OCP (Fig. 9, Supplemental data Fig. S6). These findings are consistent with results from a recent study which showed that forming enamel in *Mmp20*^−/−^ mice contains a mixture of hydroxyapatite and OCP^44^.

From Levels 2.8 through 3.0 a third layer of dysplastic enamel is formed in *Mmp20*^−/−^ mouse incisors (Fig. 5D–G; Appendix Fig. S1). This pathological layer does not form by the extension of mineral ribbons, but by the deposition of amorphous mineral possibly facilitated by rapid secretion and surface pooling of kallikrein-related peptidase-4 (KLK4) cleaved enamel proteins deposited from secretory granules (Fig. 5E,F). This mineralized layer does not form in *Amelx*^−/−^ mice (Supplemental data Fig. S2), suggesting that amelogenins play an important role during the formation of this third mineralized layer in *Mmp20*^−/−^ mice. SAED analyses indicated that the disorganized mineral phase present in the outer layer of *Mmp20*^−/−^ mice is likely hydroxyapatite (Fig. 8, top row; Fig. 9, and Supplemental data Fig. S3).

### Late secretory and early maturation stage differences between ***Amelx***^−/−^ and ***Mmp20***^−/−^ mice

**Figure 10:**
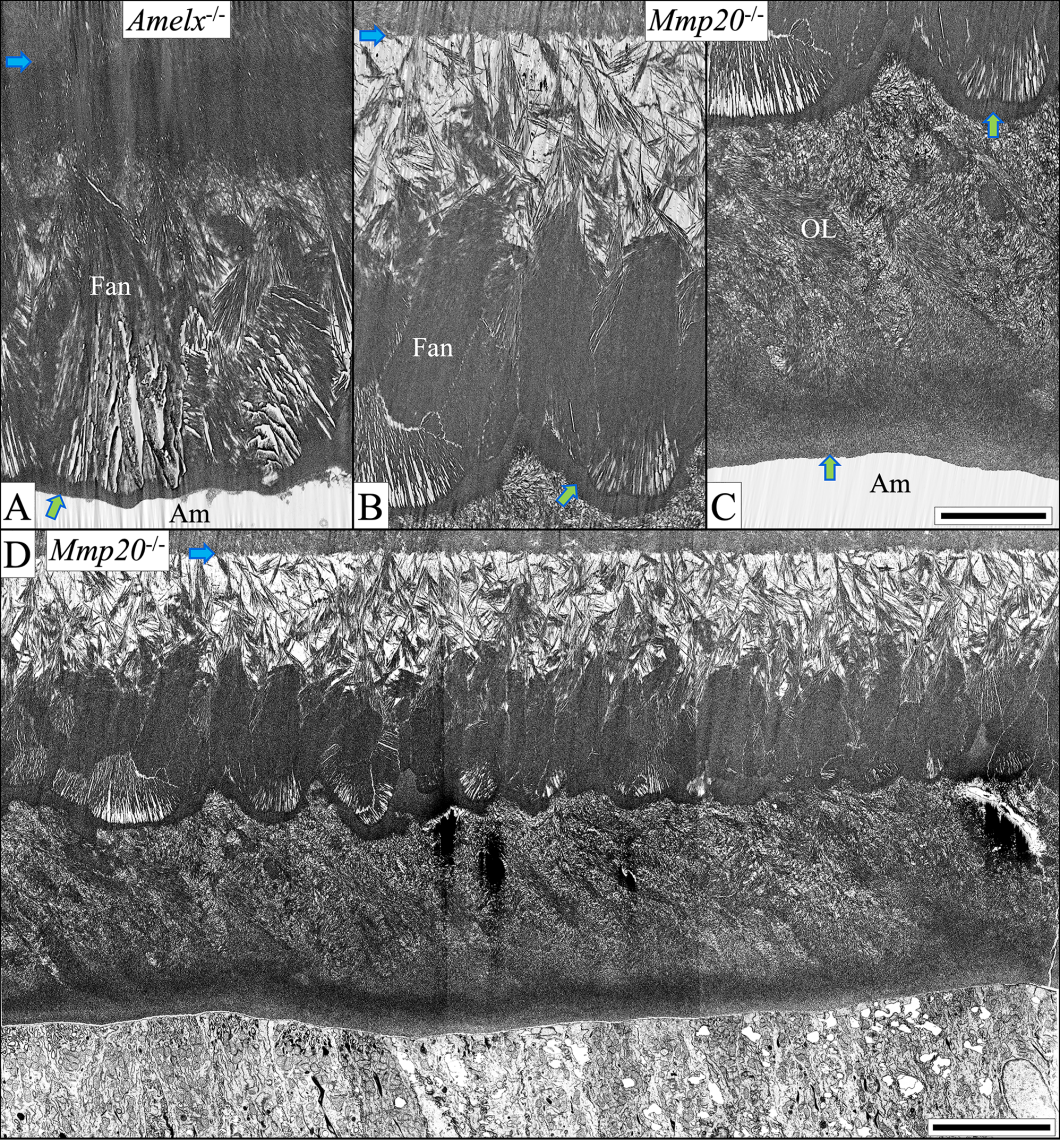
Mid maturation (Level ~ 5) in *Amelx*^−/−^ (**A**) and *Mmp20*^−/−^ (**B–D**) mice. In *Amelx*^−/−^ (**A**) and *Mmp20*^−/−^ (**B**) mice, multiple fans of varying sizes and orientations merge. Sometimes the branches of one fan appear to cross through another (like teepee fingers or Venus fly trap marginal teeth), with enlarged spaces between the stave tips. Green arrows mark a homogeneous granular mineral layer covering the tips of *Amelx*^−/−^ and *Mmp20*^−/−^ mineral fans and surface of the *Mmp20*^−/−^ outer layer. Hypomineralization atop the *Mmp20*^−/−^ DEJ (blue arrows) becomes more obvious with increased mineralization of the fans (**B**). The outer layer (OL, panel **C**) is pleomorphic and disorganized, with diagonal tracts thought to trace the retrograde movement of individual ameloblasts. Black streaks are artifacts. Ameloblasts show increasing cytoplasmic vacuolization as development progresses (**D**). Bar in (**A***–***C**) = 5 µm, in (**D**) = 10 µm.

At the end of secretory stage, WT mouse incisor enamel is around 120 µm in thickness and is comprised of the initial enamel, an inner enamel layer of decussating enamel rods angled ~ 42˚ from the horizontal plane of the DEJ, an outer layer (~ 20 µm) with the rods angled incisally ~ 20˚, and a very thin final layer of interrod enamel on top of the outer enamel layer (Fig. 8A). The dysplastic enamel in *Amelx*^−/−^ mice achieves only about one sixth of normal thickness (20–24 µm; Fig. 8B,D). The tips of the OCP mineral fans become covered by a thin granular mineralized layer as the early maturation stage begins (Fig. 8B,D; green arrows). Little change occurs thereafter except for an increase in mineral density on the outer aspects of the OCP fans (Fig. 10A).

The surface of the tips of the *Mmp20*^−/−^ fans are similarly covered by a thin granular mineralized layer (Fig. 8C,E). Subsequently, a third layer forms on top of the OCP fans (Fig. 10B–D) that is poorly organized and increases the overall dysplastic enamel thickness by ~ 15–20 µm to a total thickness of ~ 40 µm (Figs. 5C,D, 8C, 10C,D). This outer layer is not comprised of oriented mineral crystals, but is poorly structured and its pathological formation is highlighted by its abnormal ion composition^45^. Nevertheless, SAED analyses indicate that the mineral phase present is not OCP, as present in the fans, but likely hydroxyapatite (Fig. 8, top row box 5 for fans and box 6 for outer layer; (see Supplemental data on SAED pattern matching). An interesting observation in the *Mmp20*^−/−^ outer layer is the occasional appearance of a less-dense area that that is shaped and oriented like an enamel rod (Fig. 8C), and appears to be deposited during the retrograde movement of a single ameloblast (Fig. 8C). The mineral in the outer layer does not form by the elongation of mineral ribbons at the ameloblast membrane, but by the secretion of an amelogenin-rich matrix that hardens into mineral. When these secretions do not mix with those secreted by adjacent cells, the resulting mineral tracks the path of the retreating ameloblast, but is not comprised of oriented enamel mineral ribbons, like an enamel rod.

### Ultrastructure of ***Mmp20***^−/−^ secretory stage mouse incisor ameloblasts

**Figure 11:**
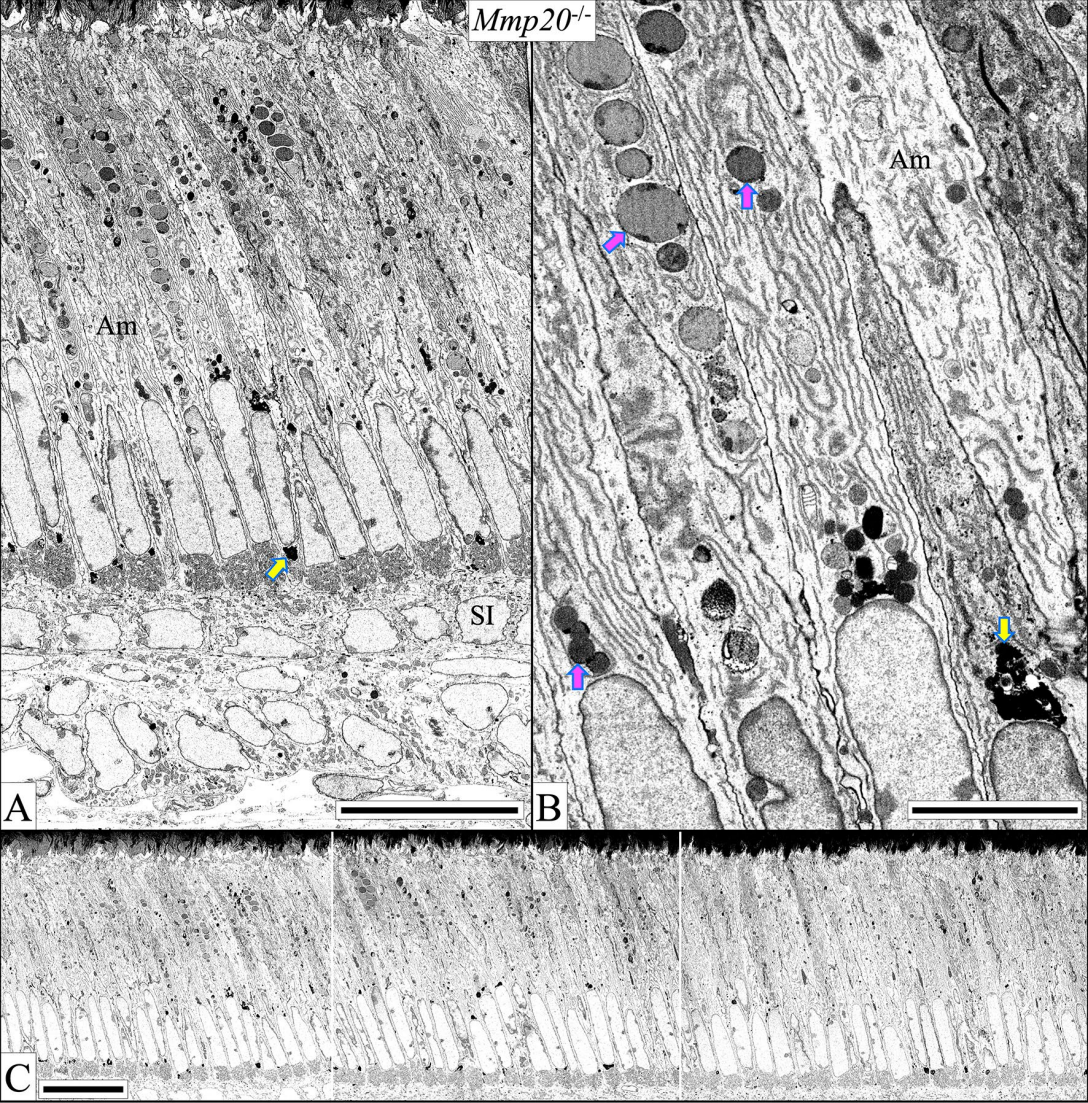
*Mmp20*^−/−^ secretory stage ameloblasts (Level 1.5–2.2) sometimes contain multiple large spherical dense bodies of varying appearances (magenta arrows) as well as more dense spherical bodies at supranuclear and infranuclear locations (yellow arrow). The quantity of these spherical bodies diminishes as the secretory stage progresses and does not disrupt the integrity or overall morphology of the ameloblast layer (**C**). These bars in (**A**) and (**C**) = 20 µm, in (**B**) = 5 µm.

**Figure 12:**
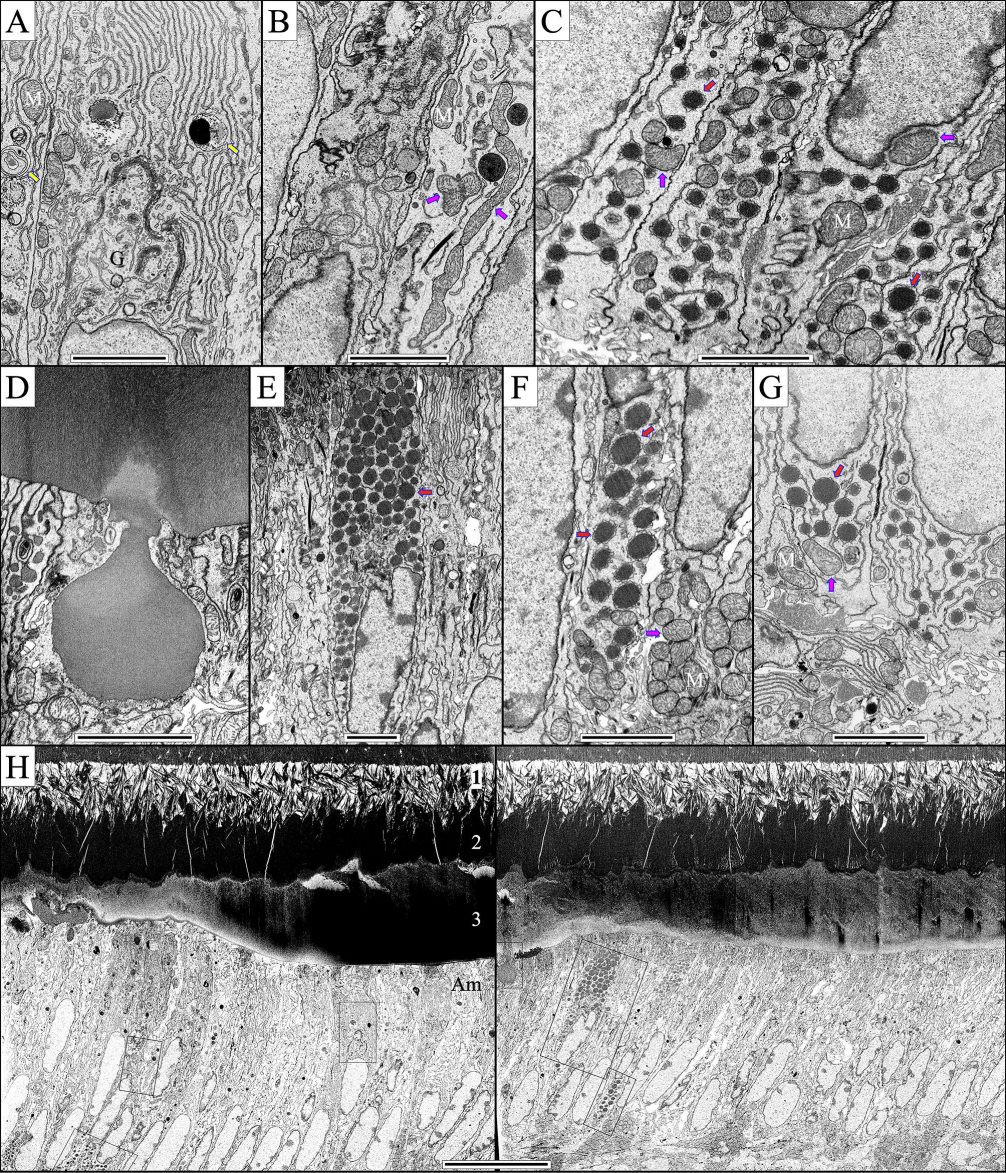
*Mmp20*^−/−^ transitional and early maturation stage ameloblasts (level 3.0–3.2) sometimes show enlarged Golgi (**A**), abundant mitochondria (magenta arrows, **B**,**C**,**F**,**G**), and numerous dense spherical supranuclear and infranuclear accumulations within bloated endoplasmic reticulum (red arrows **C**, **E***–***G**) that apparently contain secretory pathway proteins. Large surface nodule (> 3 µm in diameter) apparently formed by an accumulation of matrix secreted near the cell junctions (**D**), that may contribute to the disorganized outer mineral layer that covers the dense mineralized fan-like structures. Low magnification montages of the transitional and early maturation stage ameloblasts (**H**) mark the positions of the higher magnification views (orange boxes) and show that deposition of this outer layer does not occur by the elongation of enamel mineral ribbons, but by a pathological process involving the bulk addition of large amounts of matrix. Bars in panels (**A–G)** = 3 µm, (**H**) = 20 µm).

The most significant difference between ameloblasts from WT and *Mmp20*^−/−^ mice is the absence of a functional Tomes' processes and the presence of anomalous structures in ameloblasts. Many secretory stage ameloblasts from *Mmp20*^−/−^ mouse incisors contain enlarged lysosomes linearly arrayed between the nucleus and the distal surface, with others at supranuclear and infranuclear locations (Fig. 11). Some ameloblasts show enlarged Golgi (Fig. 12A) and abundant, large mitochondria (Fig. 12B), consistent with significant levels of protein synthesis (Fig. 12). Many ameloblasts contain swollen endoplasmic reticulum^46,47^ (Fig. 12C,E–G), and occasionally there is a granulated ameloblast^48^ (Fig. 12E). At times, secreted material that contributes to the formation of the outer layer appears to accumulate laterally or in the matrix along the cell borders (Figs. 8F, 12D). The *Mmp20*^−/−^ transition ended at Level 3.2–3.3, after which the ameloblasts showed clear vacuoles that appeared to be depleted of spherical bodies (Fig. 12H).

## Discussion

We conducted in-depth ultrastructural analyses of dental enamel formation in mouse mandibular incisors from *Amelx*^−/−^ and *Mmp20*^−/−^ mice. Montages of magnified images (1500 or 2500x) show 13 (~ 90 µm) segments of *Amelx*^−/−^ and 24 segments of *Mmp20*^−/−^ from continuously erupting mandibular incisors that were analyzed by FIB-SEM (Supplemental data Figs. S1 and S2). All early events occurred normally in the *Amelx*^−/−^ and *Mmp20*^−/−^ mice, including deposition of collagen-rich predentin, basement membrane degradation, extension of ameloblast fingerlike processes into predentin, the appearance of mineral foci in predentin near the ameloblast layer, and the coalescing of mineral islands into a continuous layer of mineralized dentin prior to enamel ribbon formation^8,9^.

An important finding was the formation of initial enamel mineral ribbons extending from the dentin surface to the ameloblast distal membrane in both *Amelx*^−/−^ and *Mmp20*^−/−^ mice. The formation of oriented enamel mineral ribbons initiating on the dentin surface and extending to the ameloblast membrane is a defining feature of dental enamel formation. As previously stated, enamel mineral ribbons do not form in mice lacking enamelin^8^ or ameloblastin^26^. Interestingly, the gar makes enamel that starts as mineral ribbons and has *Ambn* and *Enam* genes, but has no amelogenin gene^49,50^. Therefore, neither amelogenin nor MMP20 likely play a role in initial enamel ribbon nucleation, shaping or orientation in vivo, as well as when this process continues a short distance away from the ameloblast membrane to provide overall lengthening of enamel crystallites through appositional growth of the enamel layer.

Even so, amelogenin comprises ~ 90% of enamel matrix protein^51^ and is a critical enamel protein. Without amelogenins, the initially oriented murine mineral ribbons prematurely increase in density and thickness when they are only a few micrometers long. By mid-secretory stage (Level 2) the *Amelx*^−/−^ initial enamel is already more highly mineralized than dentin. The similarities between the dysplastic enamel formed in the second mineral layer of the *Amelx*^−/−^ and *Mmp20*^−/−^ mice, which both feature the formation of distinctive OCP fans terminating 20–24 µm above the dentin surface, is truly extraordinary. Since *Mmp20*^−/−^ mouse molars express normal levels of enamel matrix proteins, the only common deficit between the *Amelx*^−/−^ and *Mmp20*^−/−^ mice is the absence of amelogenin cleavage products.

Previously it was shown that when the most abundant mouse amelogenin splice product (M180) was expressed in the *Amelx*^−/−^ background, removing MMP20 reduced enamel hardness and quality of the enamel architecture, suggesting that amelogenin cleavage products might be necessary for proper enamel formation^52^. Furthermore, physical and electron microscopy studies have demonstrated that enamel protein cleavage products are different from their original secretory forms and may show distinct spatial distribution patterns within the enamel layer^53^. Since the present FIB-SEM study demonstrated that OCP fans form in the absence of amelogenin and in the absence of MMP20 (neither have amelogenin cleavage products), this is the first study to specifically implicate amelogenin cleavage products as important for proper hydroxyapatite formation and presents in vivo evidence that MMP20 is not simply degrading enamel proteins, but serves a major role by processing amelogenins into cleavage products that serve important secondary functions.

After formation of the initial enamel fan-like stems in *Amelx*^−/−^ and *Mmp20*^−/−^ mice, it appeared as though the ameloblasts lost their ability to transport secreted ions onto the tips of the existing mineral ribbons so that they would mostly elongate rather than thicken into fan-like structures. Or perhaps the environmental conditions favored the direct formation of OCP fan structures. Regardless, the fan structures appeared to occur at about the same time in *Amelx*^−/−^ and *Mmp20*^−/−^ mice. This anomalous mineralization continued in the same way in *Amelx*^−/−^ and *Mmp20*^−/−^ mice and was therefore independent of enamelin and ameloblastin proteolytic processing. Although this occurred in an atypical environment, it may suggest that the most significant function of MMP20 is the processing of amelogenins so that the enamel mineral ribbons can reach their full extension and covert into HAP at an appropriate time.

A notable difference between the *Amelx*^−/−^ and *Mmp20*^−/−^ mice was the formation of an outer disordered HAP layer exclusively in *Mmp20*^−/−^ enamel. The absence of this layer in *Amelx*^−/−^ mice indicates that the expression of amelogenin, and perhaps its KLK4 cleavage products, are critical for its formation in *Mmp20*^−/−^ mice. The outer layer was previously evident in *Mmp20*^−/−^ mice^28,45^, but was comprised mostly of surface nodules^54^. Before this study, we bred the *Amelx*^−/−^ and *Mmp20*^−/−^ mice back into the C57BL/6 background, which may account for this difference. Our ultrastructural analyses demonstrate that the *Mmp20*^−/−^ outer HAP layer does not form by the elongation of mineral ribbons along the secretory surface of the ameloblast membrane. Although it is unstructured, it can become highly mineralized.

From interpretation of these data a new theory of dental enamel formation can be deduced. First, a feedback mechanism to downregulate enamel matrix protein transcription when the forming enamel is flush with proteins likely does not exist. Second, amelogenin MMP20–mediated cleavage products, but not uncleaved amelogenins, are likely responsible for preventing development of OCP-based fan structures. Cleaved amelogenins may also be directly or indirectly responsible for successful Tomes’ process formation necessary for the development of rod and interrod areas within the developing enamel. Third, enamel mineral ribbons initiate and elongate to form the initial enamel in the total absence of amelogenin or MMP20, and as a consequence, the absence of enamelin and ameloblastin cleavage products. Overall, this study brings to light several mechanisms of enamel formation, such as the important role of amelogenin cleavage products, that provide new insights into the molecular mechanisms of dental enamel formation.

## Supplementary Information

Supplementary Information.
