# Supplementary material for: MMP20-generated amelogenin cleavage products prevent formation of fan-shaped enamel malformations
Source: Sci Rep. 2021 May 19;11:10570. doi: 10.1038/s41598-021-90005-z (PMC8134442; doi:10.1038/s41598-021-90005-z)
Supplement: Supplementary file 1 — Supplementary Information. [file 41598_2021_90005_MOESM1_ESM.docx]

**MMP20-generated Amelogenin Cleavage Products Prevent Formation of Fan-shaped Enamel Malformations**

John D. Bartlett^1^, Charles E. Smith^2,3^, Yuanyuan Hu^2^, Atsushi Ikeda^1^, Mike Strauss^3^, Tian Liang^2^, Ya-Hsiang Hsu^4,5^, Amanda H. Trout^4,5^, David W. McComb^4,5^,

Rebecca C. Freeman^2^, James P. Simmer^2*^ and Jan C-C. Hu^2^

^1^Division of Biosciences, Ohio State University College of Dentistry, Columbus, OH, USA

^2^Department of Biologic and Materials Science, University of Michigan School of Dentistry, Ann Arbor, MI, USA

^3^Department of Anatomy & Cell Biology, Faculty of Medicine & Health Sciences, McGill University, Montreal, Quebec, Canada

^4^Department of Materials Science and Engineering, Ohio State University College of Engineering, Columbus, OH, USA

^5^Center for Electron Microscopy and Analysis, Ohio State University, Columbus, OH, USA

**Supplemental Data**

**Fig. S1.** Low magnification montages of 24 FIB-bSEM runs of mouse *Mmp20*^-/-^ mandibular incisors from the apical loop to Level 4.5.

**Fig. S2.** Low magnification montages of 13 FIB-bSEM runs of mouse *Amelx*^-/-^ mandibular incisors from the apical loop to Level 4.2.

**Fig. S3.** Wild-type Outer Enamel and *Mmp20*^-/-^ Outer Enamel d-spacing Matches that of Hydroxyapatite.

**Fig. S4.** Wild-Type Dentin, Outer, Middle, and Inner Enamel Diffraction Results.

**Fig. S5.** *Amelx*^-/-^ and *Mmp20*^-/-^ Enamel Diffraction Results.

**Fig. S6.** Model References Match *Amelx*^-/-^ Enamel and *Mmp20^-/-^* Fan Enamel to OCP.


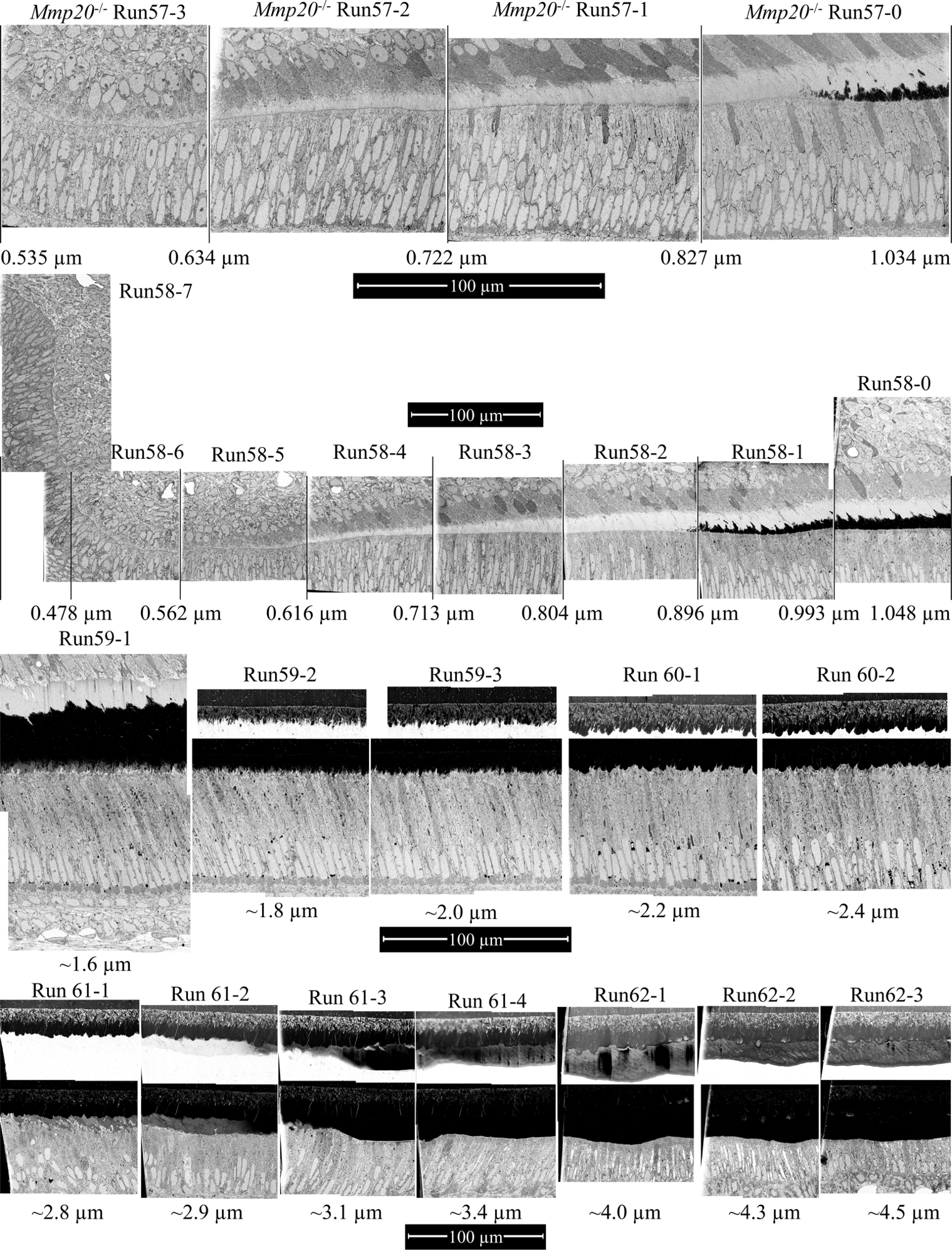


**Fig. S1.** Low magnification montages of 24 FIB-bSEM of mouse *Mmp20*^-/-^ mandibular incisors from the apical loop to Level 4.5. These montages were assembled prior to the application of descratching software.


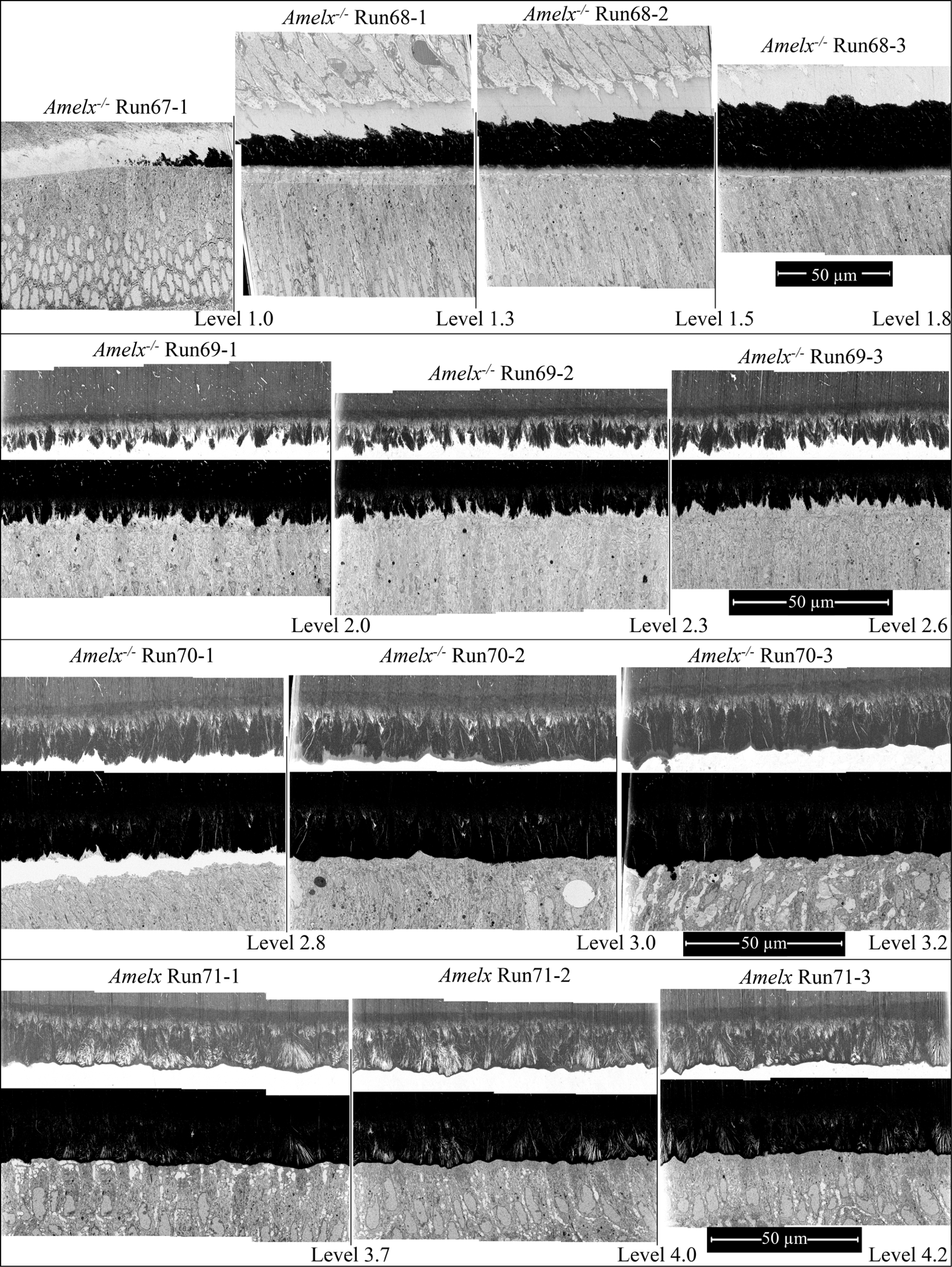


**Fig. S2.** Low magnification montages of 13 FIB-bSEM runs of mouse *Amelx*^-/-^ mandibular incisors from the apical loop to Level 4.2. These montages were assembled prior to the application of descratching software.

Calculated Hydroxyapatite WT Enamel - outer region *Mmp20*^-/-^ Enamel - outer layer

(Box 2 in Fig. 8A/B) (Box 8 in Fig. 8A/B)

**
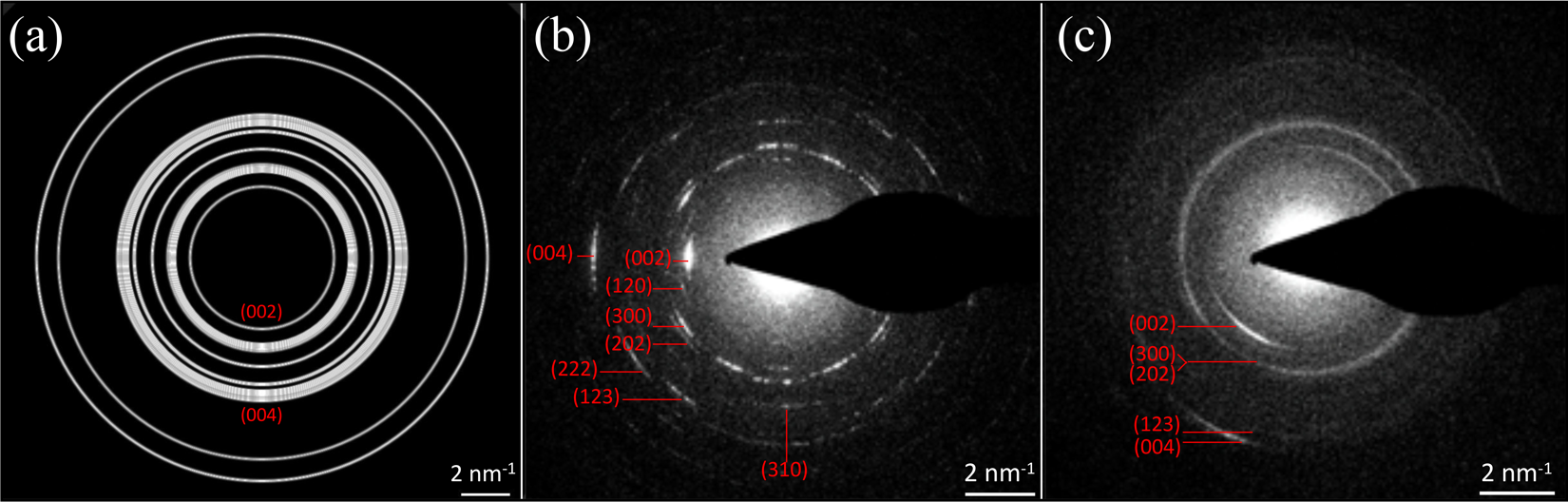
**

**Fig. S3. Wild-type Outer Enamel and *Mmp20*^-/-^ Outer Enamel d-spacing Matches that of Hydroxyapatite.** The calculated electron diffraction pattern of (a) hydroxyapatite (ICSD-26204) used as a reference standard for the experimental SAED patterns acquired from (b) WT Enamel-outer obtained from box 2 in Fig. 8A/B and (c) *Mmp20*^-/-^ Enamel-outer layer obtained from box 8 in Fig. 8A/D. The higher intensity diffraction spots/rings have been labeled. The table below provides the d-spacing values and the calculated ratios of d-spacing values to eliminate errors in calibration. hkl denotes the Miller indices for the reflections. The ratios were calculated to (002). Analysis of these electron diffraction patterns support the conclusion that both wild-type outer enamel and *Mmp20*^-/-^ outer enamel layers contain hydroxyapatite and/or components that have similar lattice parameters as hydroxyapatite, such as carbonated hydroxyapatite.

**Fig. S4a. (a) Wild-Type Dentin, (b) Outer, (c) Middle, and (d) Inner Enamel Diffraction Results.** Shown are electron diffraction patterns from a sagittally sectioned wild-type 7-week mouse mandibular incisor labeling the possible hkl indices for the stronger reflections corresponding to hydroxyapatite (ICSD-26204). These diffraction patterns are larger versions of the same images shown in Fig. 8A, boxes 1-4. The unlabeled images are reproduced in Fig. S4b (below).

**Fig. S4b.** Unlabeled SAED images from Wild-Type Dentin (upper left; box 1 Fig. 8A), Outer (upper right; box 2 Fig. 8A), Middle (lower left; box 3 Fig. 8A), and Inner Enamel (lower left; box 4 Fig. 8A).


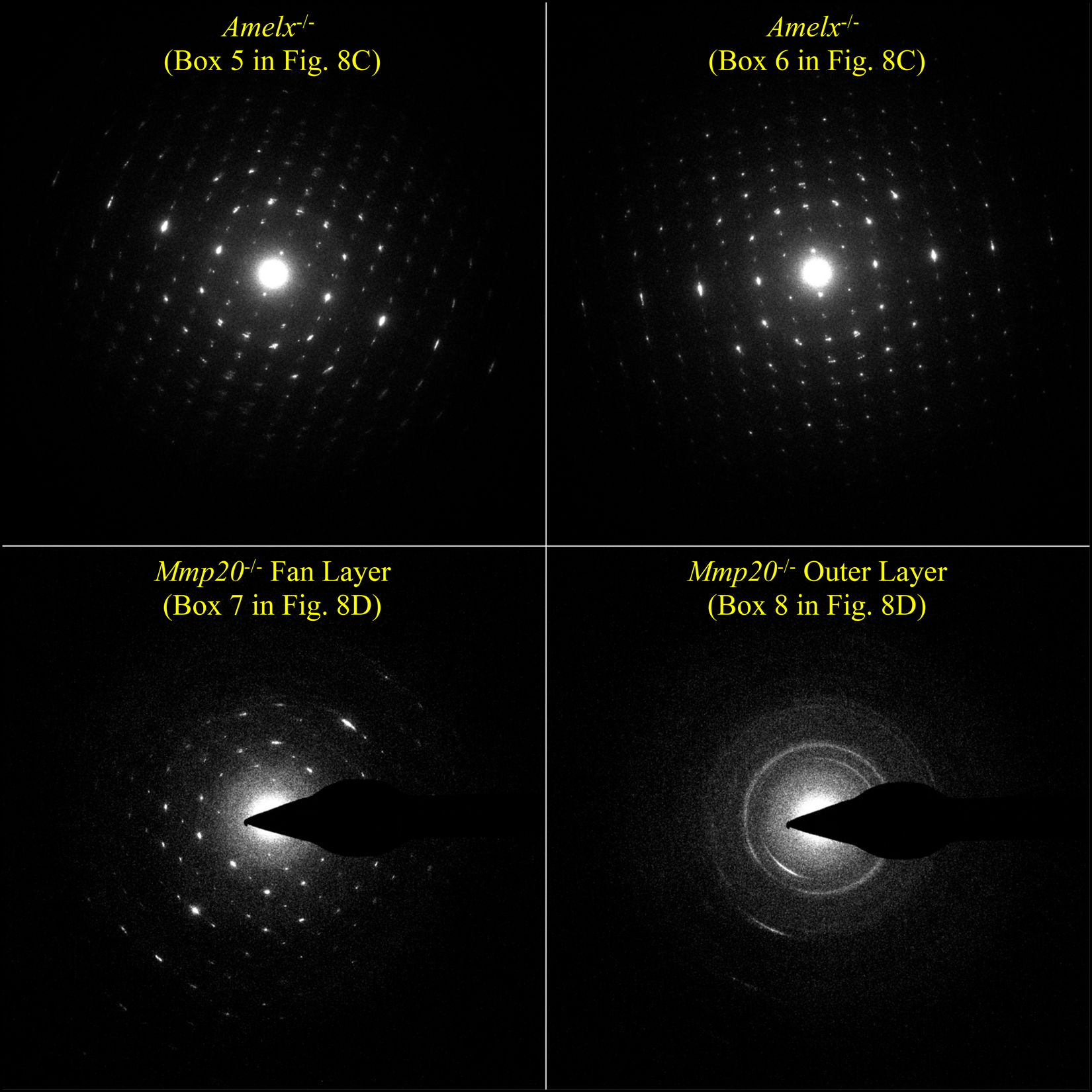


**Fig. S5. *Amelx*^-/-^ and *Mmp20*^-/-^ Enamel Diffraction Results.** Shown are electron diffraction patterns from sagittally sectioned *Amelx*^-/-^ (top) and *Mmp20*^-/-^ (bottom) 7-week mouse mandibular incisors. These diffraction patterns are larger versions of the same images shown in Fig. 8A boxes 5-8.

**Fig. S6. Model References Match *Amelx*^-/-^ Enamel and *Mmp20^-/-^* Fan Enamel to OCP.** Hydroxyapatite (b) (ICSD-26204), calcium polyphosphate (c) (ICSD-60117), monoclinic hydroxyapatite (d) (ICSD-34457), and octacalcium phosphate (e) (ICSD-65347) in red overlaid on the diffraction pattern from *Amelx^-/-^* enamel (a). Although the diffraction pattern of monoclinic hydroxyapatite can match, some of the forbidden spots (indicated in the blue rectangle), which do not appear in the simulation, are evident in the *Amelx^-/-^* diffraction image. Except for octacalcium phosphate, the other mineral phases do not match the diffraction pattern of the *Amelx^-/-^* fans. Octacalcium phosphate also matches the diffraction pattern of *Mmp20^-/-^* fan as shown in (f) with the octacalcium phosphate (ICSD-65347) pattern overlaid on the *Mmp20^-/-^* fan SAED. The table at the bottom contains the comparison of the d-spacing values and ratios to (002).This analysis indicates that the fan structures in both null mice have a similar mineral component phase, which also closely matches that of octacalcium phosphate.
